# Oscillatory Behaviors of microRNA Networks: Emerging Roles in Retinal Development

**DOI:** 10.3389/fcell.2022.831750

**Published:** 2022-02-02

**Authors:** Elizabeth S. Fishman, Jisoo S. Han, Anna La Torre

**Affiliations:** Department of Cell Biology and Human Anatomy, University of California, Davis, Davis, CA, United States

**Keywords:** MiR-183 cluster, Let-7, miR-9, circadian rhythm, cell cycle, Notch

## Abstract

A broad repertoire of transcription factors and other genes display oscillatory patterns of expression, typically ranging from 30 min to 24 h. These oscillations are associated with a variety of biological processes, including the circadian cycle, somite segmentation, cell cycle, and metabolism. These rhythmic behaviors are often prompted by transcriptional feedback loops in which transcriptional activities are inhibited by their corresponding gene target products. Oscillatory transcriptional patterns have been proposed as a mechanism to drive biological clocks, the molecular machinery that transforms temporal information into accurate spatial patterning during development. Notably, several microRNAs (miRNAs) -small non-coding RNA molecules-have been recently shown to both exhibit rhythmic expression patterns and regulate oscillatory activities. Here, we discuss some of these new findings in the context of the developing retina. We propose that miRNA oscillations are a powerful mechanism to coordinate signaling pathways and gene expression, and that addressing the dynamic interplay between miRNA expression and their target genes could be key for a more complete understanding of many developmental processes.

## Introduction

The surge of new techniques to survey the transcriptome over the last few decades has led to the identification of numerous types of non-coding RNAs. While protein-coding sequences constitute less than 1.5% of the human genome, large-scale screenings have revealed that virtually the entire genome is transcribed to generate myriads of non-coding RNAs (Kapranov et al., 2002; Carninci et al., 2005; Mattick and Makunin, 2006; Birney et al., 2007). These RNA molecules are differentially expressed in distinct cell types and dynamically regulated during development (Hangauer et al., 2013; Mercer and Mattick, 2013).

Among non-protein coding RNAs, microRNAs (miRNAs) have emerged as key post-transcriptional regulators of gene expression (Bushati and Cohen, 2007; Bartel, 2009; Chekulaeva and Filipowicz, 2009). MiRNAs are small (∼22-nucleotide (nt) long), evolutionarily conserved molecules. First described in *Caenorhabditis elegans* (Lee et al., 1993), miRNAs are also present in a wide diversity of organisms in the bacteria, archaea, and eukaryote domains (Dexheimer and Cochella, 2020).

**FIGURE 1:**
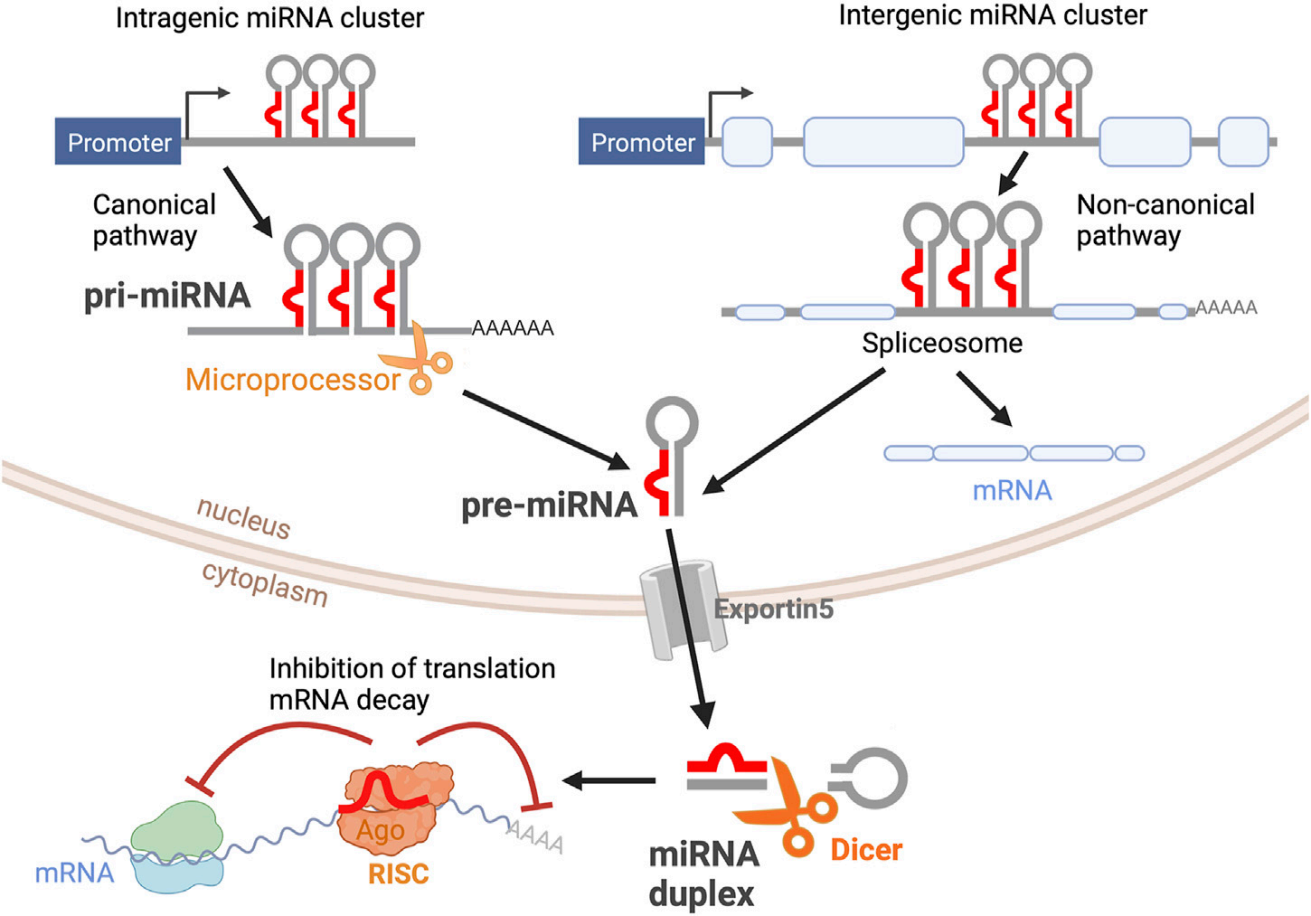
miRNA biogenesis. Primary miRNAs (pri-miRNAs) are transcribed as double-stranded hairpin-like structures. Intragenic pri-miRNAs are processed *via* the canonical pathway, where the clusters of hairpin-like structures are cleaved into individual precursor miRNAs (pre-miRNAs) by the microprocessor complex. Intergenic pri-miRNAs are processed by the splicing machinery. The resultant pre-miRNA from both pathways is an individual hairpin-like structure of 70-nt. After being exported from the nucleus *via* Exportin5, pre-miRNAs are further processed by Dicer into mature miRNA duplexes. One miRNA strand is loaded into the RNA-Induced Silencing Complex (RISC). Mature miRNA binds to its target mRNA, inhibiting mRNA translation and inducing mRNA decay. This figure was created with BioRender.com.

MiRNAs are transcribed from DNA sequences as long transcripts called primary miRNAs (pri-miRNAs) that contain double-stranded hairpin-like structures in which at least one of the two strands includes a mature miRNA (Figure 1). About half of all currently identified miRNAs are intergenic, mostly localized in introns, and controlled by the regulatory elements of the host gene; the other half are intragenic and are regulated independently by their own promoters (Ha and Kim, 2014). About 25% of all miRNAs are arranged in clusters and transcribed as longer transcripts that contain more than one mature miRNA sequence. Intergenic miRNAs are processed by the splicing machinery, while intragenic pri-miRNAs are cleaved by the microprocessor complex that includes Drosha ribonuclease and DiGeorge critical region 8 (DGCR8). In both cases, this first cleavage step produces a precursor miRNA (pre-miRNA) of about 70-nt that is exported out of the nucleus. Pre-miRNAs are further processed by the enzyme Dicer, which removes the loop of the hairpin, yielding a mature miRNA duplex that can be loaded onto the RNA-Induced Silencing Complex (RISC, Figure 1). Mature miRNAs bind to their target mRNAs, usually to the 3′ untranslated region (3′UTR), through imperfect base-pairing, hindering the stability and translation of their target mRNAs (Eulalio et al., 2008). Hence, miRNAs are part of complex networks where one individual miRNA can regulate a large number of genes, frequently from a similar biochemical pathway, and where a single target mRNA can be regulated concomitantly by multiple miRNAs. Thus far, about 2,500 mature miRNAs have been identified in the human genome (miRBase.org) (Kozomara and Griffiths-Jones, 2011), and bioinformatics studies have estimated that over 60% of the human transcriptome is regulated by miRNAs (Friedman et al., 2009).

A large body of research suggests that this previously unknown miRNA-based regulation is crucial for many physiological and pathophysiological events and that the complex interactions between transcription factors and miRNAs could be instrumental in delineating developmental programs.

### miRNAs in the Developing Retina

To gain further understanding of the roles of miRNAs in ocular tissues, several groups have attempted to characterize the retina miRNome by *in situ* hybridization, computational predictions, and profiling techniques. Hundreds of different miRNAs have been identified in the retina of different species (Loscher et al., 2007; Xu et al., 2007; Bak et al., 2008; Hackler et al., 2010; Karali et al., 2010, 2016; Fishman et al., 2021) and several miRNAs show a significantly enriched expression in the retina compared to other tissues (Table 1). Two early reports by Hackler *et al.* (Hackler et al., 2010) and Xu *et al.* (Xu et al., 2007) compared miRNA expression patterns at different developmental ages in the mouse retina and brain. Consistent with other studies (Lewis et al., 2003), the authors found that miRNAs with identical seed sequences exhibited highly similar expression profiles. Additionally, these studies and others have defined the repertoire of miRNAs expressed at different time points during retinal development (Table 1). Two main miRNA categories have been consistently identified: miRNAs expressed primarily at early developmental stages (embryonic day (E)10-E16 in the mouse) and miRNAs present at later stages of retinal development (E16-postnatal day (P)7) and maturation (>P7). Specifically, miR-17, miR-18, miR-19, miR-20, miR-93, miR-106, and miR-130 are down-regulated throughout development, while the let-7 family, miR-7, miR-9, miR-9*, miR-96, miR-101, miR-124, miR-181, miR-182, and miR-183 are some of the miRNAs that increase during retinal development from E10 to adulthood in mice. Additional studies have also identified cell-specific expression of subsets of these miRNAs (Georgi and Reh, 2010; Ohana et al., 2015; Wohl and Reh, 2016a) (Table 1).

**TABLE 1:** Summary of miRNAs highly expressed in the developing retina.

miRNA	Enriched in retina	Development expression	Known function	Proposed roles in retinal diseases	References
Let-7a	no	late development	neural differentiation; competence progression, repression of regeneration	Retinoblastoma	(Arora et al., 2007; Bak et al., 2008; Georgi and Reh, 2010; Hackler et al., 2010; Mu et al., 2010; Ramachandran et al., 2011; La Torre et al., 2013; Xia and Ahmad, 2016; Fairchild et al., 2019)
Let-7b	no	late development	neural differentiation; competence progression, repression of regeneration	Retinoblastoma, diabetic retinopathy	(Makarev et al., 2006; Bak et al., 2008; Hackler et al., 2010; Mu et al., 2010; Georgi and Reh, 2011; Xia and Ahmad, 2016; Fairchild et al., 2019; Smit-McBride et al., 2020)
Let-7c	no	late development	neural differentiation; competence progression, repression of regeneration	AMD, Retinoblastoma	(Bak et al., 2008; Hackler et al., 2010; Mu et al., 2010; Georgi and Reh, 2011; Ertekin et al., 2014; Xia and Ahmad, 2016; Fairchild et al., 2019)
Let-7d	no	late development	neural differentiation; competence progression, repression of regeneration	Retinobastoma	(Bak et al., 2008; Hackler et al., 2010; Mu et al., 2010; Georgi and Reh, 2011; La Torre et al., 2013; Fairchild et al., 2019)
Let-7e	no	late development	neural differentiation; competence progression, repression of regeneration	Retinobastoma	(Bak et al., 2008; Hackler et al., 2010; Mu et al., 2010; Georgi and Reh, 2011)
Let-7f	no	late development	neural differentiation; competence progression, repression of regeneration	Retinobastoma	(Ryan et al., 2006; Bak et al., 2008; Hackler et al., 2010; Georgi and Reh, 2011; Ramachandran et al., 2011; La Torre et al., 2013)
miR-101a		late development			(Arora et al., 2007; Georgi and Reh, 2011)
miR-103		late development			(Xu et al., 2007; Fishman et al., 2021)
miR-106	yes	early development		AMD	(Xu et al., 2007; Hackler et al., 2010; La Torre et al., 2013; Ertekin et al., 2014)
miR-107		early development			(Arora et al., 2007; Xu et al., 2007; Hackler et al., 2010)
miR-124	no	late development	neuronal fate determination	AMD and other neurodegenerations	(Deo et al., 2006; Makarev et al., 2006; Ryan et al., 2006; Arora et al., 2007; Loscher et al., 2007; Xu et al., 2007; Bak et al., 2008; Qiu et al., 2009; Karali et al., 2011; Chu-Tan et al., 2018; Wohl et al., 2019)
miR-125b	no	late development/ no change	Competence progression neuronal differentiation	AMD, Retinoblastoma	(Makarev et al., 2006; Ryan et al., 2006; Loscher et al., 2007; Bak et al., 2008; Georgi and Reh, 2010; Hackler et al., 2010; Yang and Mei, 2015; Berber et al., 2017)
miR-127	no	late development			(Xu et al., 2007; Hackler et al., 2010)
miR-128a	no				(Xu et al., 2007; Bak et al., 2008)
miR-129		early development	photoreceptor/bipolar fate		(Decembrini et al., 2009)
miR-139					(Arora et al., 2007; Xu et al., 2007)
miR-140	yes				(Arora et al., 2007; Xu et al., 2007)
miR-15a		downregulated postnatally			(Wohl and Reh, 2016a)
miR-15b		enriched in fovea, downregulated postnatally		Diabetic retinopathy	(Wohl and Reh, 2016a; Wang et al., 2016; Fishman et al., 2021)
miR-151	yes				(Xu et al., 2007)
miR-155		early development	photoreceptor/bipolar fate	AMD	(Decembrini et al., 2009)
miR-16	no	early development			(Arora et al., 2007; Georgi and Reh, 2010; Hackler et al., 2010)
miR-17	no	early development	Retinal progenitor proliferation, circadian oscillator regulator	AMD, Retinoblastoma	(Arora et al., 2007; Hackler et al., 2010; Georgi and Reh, 2011; Sage and Ventura, 2011; Wohl and Reh, 2016a; Gao et al., 2016b; Berber et al., 2017)
miR-18	no	early development		Retinoblastoma	(Hackler et al., 2010; Georgi and Reh, 2011; Yang and Mei, 2015)
miR-181a		late development		Glaucoma, LHON	(Arora et al., 2007; Loscher et al., 2007; Hackler et al., 2010; Karali et al., 2016; Indrieri et al., 2019)
miR-181b		late development		Glaucoma, LHON	(Lagos-Quintana et al., 2003; Wienholds et al., 2005; Makarev et al., 2006; Ryan et al., 2006; Arora et al., 2007; Xu et al., 2007; Indrieri et al., 2019)
miR-181c	yes	late development			(Arora et al., 2007; Xu et al., 2007; Hackler et al., 2010)
miR-182	yes	Enriched in photoreceptors	photoreceptor physiology, circadian oscillator regulator		(Lagos-Quintana et al., 2003; Wienholds et al., 2005; Ryan et al., 2006; Arora et al., 2007; Loscher et al., 2007; Xu et al., 2007; Bak et al., 2008; Hackler et al., 2010; Georgi and Reh, 2011; Lumayag et al., 2013; Busskamp et al., 2014; Karali et al., 2016; Fogerty et al., 2019; Fairchild et al., 2021; Fishman et al., 2021)
miR-183	yes	Enriched in photoreceptors	photoreceptor physiology, circadian oscillator regulator	RP	(Lagos-Quintana et al., 2003; Wienholds et al., 2005; Ryan et al., 2006; Arora et al., 2007; Loscher et al., 2007; Xu et al., 2007; Bak et al., 2008; Hackler et al., 2010; Georgi and Reh, 2011; Lumayag et al., 2013; Busskamp et al., 2014; Karali et al., 2016; Fogerty et al., 2019; Fairchild et al., 2021; Fishman et al., 2021)
miR-184	yes			AMD	(Lagos-Quintana et al., 2003; Loscher et al., 2007; Xu et al., 2007; Bak et al., 2008; Karali et al., 2016; Intartaglia et al., 2020)
miR-185	yes				(Lagos-Quintana et al., 2003; Xu et al., 2007)
miR-191					(Georgi and Reh, 2011)
miR-194	yes				(Xu et al., 2007)
miR-200b*				AMD, Diabetic retinopathy, Glaucoma	(Georgi and Reh, 2011; Gao et al., 2016a; Dantas da Costa et al., 2019)
miR-204			retina and lens development	AMD, Coloboma, Glaucoma	(Wienholds et al., 2005; Deo et al., 2006; Ryan et al., 2006; Xu et al., 2007; Conte et al., 2010; Villarreal et al., 2011; Karali et al., 2016; Intartaglia et al., 2020)
miR-21	no	late development		AMD	(Makarev et al., 2006; Hackler et al., 2010)
miR-210	yes				(Xu et al., 2007; Bak et al., 2008; Hackler et al., 2010)
miR-211	yes				(Arora et al., 2007; Xu et al., 2007; Bak et al., 2008)
miR-214		early development	photoreceptor/bipolar fate		(Decembrini et al., 2009)
miR-219	yes				(Arora et al., 2007; Xu et al., 2007)
miR-222		early development	photoreceptor/bipolar fate		(Decembrini et al., 2009)
miR-24a		late development	inhibition of apoptosis	AMD, Glaucoma	(Walker and Harland, 2009; Intartaglia et al., 2020)
miR-25	yes	downregulated postnatally	circadian oscillator regulator	Retinoblastoma	(Arora et al., 2007; Xu et al., 2007; Yang and Mei, 2015; Wohl and Reh, 2016a)
miR-26a	yes		circadian oscillator regulator	AMD	(Ryan et al., 2006; Loscher et al., 2007; Georgi and Reh, 2011; Ertekin et al., 2014)
miR-29b		late development		AMD, Diabetic retinopathy, Glaucoma	(Arora et al., 2007; Dantas da Costa et al. 2019; Xu et al., 2007; Hackler et al., 2010; Villarreal et al., 2011; Intartaglia et al., 2020)
miR-29c		late development		Glaucoma	(Arora et al., 2007; Hackler et al., 2010; Karali et al., 2010; Villarreal et al., 2011)
miR-30	no	late development			(Wienholds et al., 2005; Ryan et al., 2006; Arora et al., 2007; Bak et al., 2008; Hackler et al., 2010; Georgi and Reh, 2011; Fishman et al., 2021)
miR-31	yes				(Ryan et al., 2006; Loscher et al., 2007; Xu et al., 2007; Bak et al., 2008)
miR-320	yes			Diabetic retinopathy	(Xu et al., 2007; Smit-McBride et al., 2020)
miR-342-5p		late development, enriched in peripheral/nasal retina	neural stem cell proliferation	AMD	(Ertekin et al., 2014; Gao et al., 2017; Fishman et al., 2021)
miR-361	yes				(Xu et al., 2007)
miR-550		late development			(Georgi and Reh, 2011)
miR-690		late development			(Georgi and Reh, 2011)
miR-7	no	early development			(Arora et al., 2007; Xu et al., 2007; Bak et al., 2008; Georgi and Reh, 2011)
mir-709		late development			(Georgi and Reh, 2011)
miR-720					(Georgi and Reh, 2011; La Torre et al., 2013)
miR-9/9*	yes	late development	neuronal fate determination	AMD, Macular Telangiectasia Type 2	(Wienholds et al., 2005; Deo et al., 2006; Arora et al., 2007; Loscher et al., 2007; Xu et al., 2007; Hackler et al., 2010; Georgi and Reh, 2011; La Torre et al., 2013; Thomas et al., 2021)
miR-92	yes	progenitors		Retinoblastoma	(Deo et al., 2006; Kapsimali et al., 2007; Xu et al., 2007; Sage and Ventura, 2011)
miR-93		early development			(Arora et al., 2007; Hackler et al., 2010; Georgi and Reh, 2011; Fishman et al., 2021)
miR-96	yes	late development	photoreceptor physiology, circadian oscillator regulator	RP	(Lagos-Quintana et al., 2003; Wienholds et al., 2005; Ryan et al., 2006; Arora et al., 2007; Loscher et al., 2007; Xu et al., 2007; Bak et al., 2008; Hackler et al., 2010; Georgi and Reh, 2011; Lumayag et al., 2013; Busskamp et al., 2014; Karali et al., 2016; Fogerty et al., 2019; Fishman et al., 2021)

Retina enrichment is defined as increased expression compared to brain samples; early development refers to E10-E16 and late development refers to E16-P7, as defined by the progenitor states in Clark et al. (Clark et al., 2019). Acronyms: AMD, age-related macular degeneration; LHON, Leber’s hereditary optic neuropathy; RP, retinitis pigmentosa.

**FIGURE 2:**
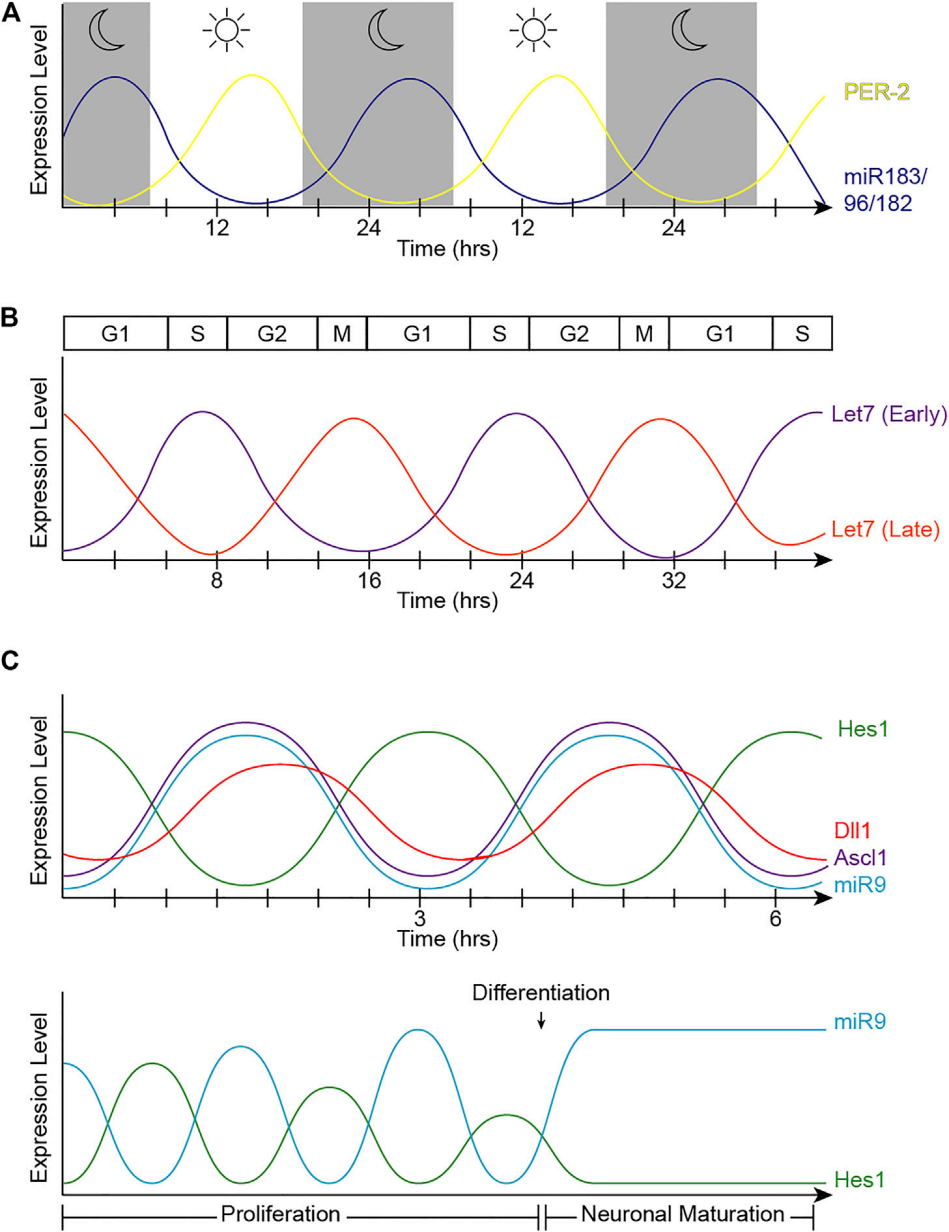
Oscillatory patterns of miRNAs in biological processes. **(A)** Oscillatory behavior of the miR-183 complex. miR-96 directly targets PER2, causing an out-of-phase oscillation pattern with PER-2 peaking during light and miR-183/96/182 peaking in dark hours. **(B)** Let-7 oscillatory behavior. The fluctuation of let-7 expression in accordance with the cell cycle changes at different stages of development. Early in development (let-7 early), let-7 expression is at its lowest at the start of the cell cycle in G1, and peaks in S-phase. The phase of oscillation shifts later in development (let-7 late), when let-7 expression is at its highest in mitosis and lowest in S-phase. **(C)** Hes1/miR-9 ultradian oscillator. (Top) Hes1 oscillation is self-driven with a rhythmicity of 2–3 h. The Hes1 oscillator represses Ascl1 and Notch ligands, consequently driving their oscillation patterns. MiR-9 and Hes1 participate in a negative feedback loop. (Bottom) Hes1 and miR-9 have out-of-phase expression patterns and are dependent on one another. As miR-9 continues to accumulate during proliferation, Hes1 is consequently dampened. RPC differentiation is induced when miR-9 levels reach a threshold to maintain high, steady levels while dulling Hes1 oscillations, resulting in neuronal maturation.

Dicer and DGCR8 transgenic models (Damiani et al., 2008; Decembrini et al., 2008; Georgi and Reh, 2010, 2011; Pinter and Hindges, 2010; Davis et al., 2011; Iida et al., 2011; La Torre et al., 2013; Busskamp et al., 2014; Sundermeier et al., 2017), miRNA mutants (Lumayag et al., 2013; Barbato et al., 2017; Fogerty et al., 2019), sponge strategies (Zhu et al., 2011), and miRNA inhibitors (Decembrini et al., 2009; La Torre et al., 2013; Taylor et al., 2019; Wohl et al., 2019) have been extensively used to shed some light on the specific roles of miRNAs during retinal development. While many miRNA functions have been elucidated using these strategies, far less is known about miRNA target genes and the specific circuits that regulate development and pathophysiological processes in the retina. Furthermore, these global analyses do not capture the dynamic nature of miRNA expression and activity. Importantly, several miRNAs are involved in complex feedback and feed-forward regulations with their target genes, allowing for increased robustness of protein expression towards gene background noise (Borenstein and Ruppin, 2006). MiRNAs also participate in negative feedback loops, where target mRNAs regulate miRNA expression, leading to the occurrence of biological rhythms. Correspondingly, miRNAs have been shown to display rhythmic behaviors in the retina and other organs, and to regulate the circadian clock, the cell cycle, and the Hes1 ultradian oscillator (Figure 2). Here, we summarize some of the recent findings on miRNA oscillatory behaviors, their regulatory mechanisms, and some of their possible functions during retinal development.

### miR-183, -96, -182 and the Circadian Clock

**FIGURE 3:**
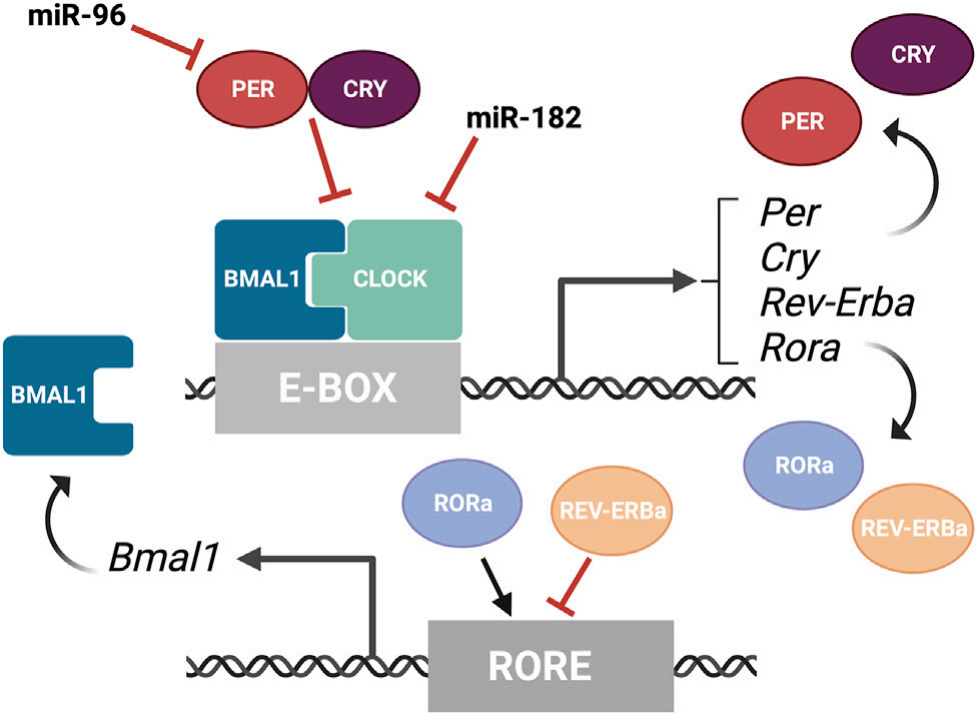
Overview of the molecular components of the circadian rhythm. Circadian rhythm is regulated by two interwoven feedback loops. The first loop involves CLOCK and BMAL1 activating regulatory elements containing E-boxes to induce expression of repressors. PER and CRY proteins bind to CLOCK/BMAL1 to repress the transcriptional activity of their activators. MiR-182 targets CLOCK, among other circadian rhythm regulators, and miR-9 targets PER-2. The second loop involves REV-ERBα and RORα competing for binding on RORE binding elements, which promotes Bmal1 transcription. This figure was created with BioRender.com.

The textbook view of the circadian clock consists of a light-dark pattern of approximately 24 h (Figure 2A) that governs rhythmicity within the organism and is regulated by two interwoven feedback loops with positive and negative components (Figure 3). One of these regulatory mechanisms involves the heterodimeric transcriptional activators CLOCK and BMAL1, which trigger the expression of repressors such as Period (PER1, PER2, and PER3) and Cryptochrome (CRY1 and CRY2) that, in turn, will repress the transcriptional activity of their activators (Sangoram et al., 1998; Zylka et al., 1998; Lowrey and Takahashi, 2004). The second loop involves the expression of Rev-Erbα and Rorα genes also regulated by CLOCK and BMAL1. Subsequently, REV-ERBα and RORα proteins compete for binding to the Bmal1 promoter (Lowrey and Takahashi, 2004). These self-sustaining feedback clocks are reset by fluctuating inputs, including light, temperature, or feeding patterns, to synchronize the molecular clock with the environment and the Earth’s rotation. This timing mechanism is controlled by a master pacemaker in the suprachiasmatic nuclei (SCN) of the hypothalamus, but independent circadian oscillators are present throughout the organism. Studies in the early 80’s already demonstrated that the circadian clock was present in the *Xenopus* retina (Besharse and Iuvone, 1983), and further analyses have added that the retinal circadian rhythm controls many aspects of the vertebrate ocular physiology, including melatonin and dopamine synthesis, photoreceptor disk shedding, visual sensitivity, and intraocular pressure (LaVail and Ward, 1978; Doyle et al., 2002; Maeda et al., 2006; Storch et al., 2007; Tosini et al., 2008). Dysregulation of these retinal circadian clocks can lead to ocular diseases and have impacts on the circadian rhythm within the whole body (Ko, 2020).

Mathematical modeling predicted decades ago that the regulation of mRNA stability is essential for rhythmic protein output (Wuarin et al., 1992). More recently, high-throughput analyses have shown that 25–50% of all rhythmically expressed proteins do not exhibit transcriptional rhythmicity (Mauvoisin et al., 2014). Accordingly, instead of the simplified transcription-translation view, the circadian rhythm undergoes very complex and dynamic regulatory processes that include polyadenylation, RNA splicing, and miRNA regulation.

Numerous miRNAs exhibit circadian rhythmicity, although the mechanisms that regulate these oscillations often remain unclear. In some cases, miRNA coding regions contain E-Box or RORE upstream elements that could be regulated by the core components of the circadian clock (Cheng et al., 2007). Dicer expression has also been reported to display diurnal rhythmicity (Yan et al., 2013), which could lead to a rhythmic pattern of miRNA maturation.

By means of microarray technologies and other tools, early screenings identified the miR-183 cluster (miR-183, miR-96, and miR-182) as miRNAs robustly regulated by the circadian clock (Xu et al., 2007; Yang et al., 2008). For instance, circadian fluctuations in dme-miR-263a and dme-miR-263b expression, the *Drosophila* orthologues of the miR-183 cluster, were detected in wild type flies and the levels of these miRNAs were significantly reduced in the arrhythmic clock mutant *cyc* ^*01*^ (Yang et al., 2008). Likewise, in the adult mouse retina, the expression of these miRNAs obeys a circadian rhythm, with the miRNA levels being significantly higher during zeitgeber time (ZT) 17 (midnight) compared to ZT 5 (noon) (Xu et al., 2007). The expression of these miRNAs is also regulated by light in the mammalian retina and the total levels of miR-183, miR-96, and miR-182 shift quickly (within 30 min) after light or dark adaptation (Krol et al., 2010).

MiR-183, -96, and -182 are part of a highly-conserved polycystronic miRNA cluster that plays multiple roles in sensory tissues including the retina (Lagos-Quintana et al., 2002; Xu et al., 2007), the inner ear (Weston et al., 2006), and the olfactory epithelium (Xu et al., 2007). In the vertebrate retina, this cluster has been shown to elicit neuroprotective functions in photoreceptors, modulate outer segment maintenance, and enhance light responses in stem cell-derived retinal organoids (Loscher et al., 2007; Krol et al., 2010; Lumayag et al., 2013; Busskamp et al., 2014). Many reports indicate that the miR-183 cluster is a key regulator of apoptosis and programmed cell death and validated target genes include CASP2, FOXO1, SLC1A1, and PDCD4 (Zuzic et al., 2019). Recent studies have indicated that the miR-183 cluster is also an important morphogenetic factor regulating multiple signaling pathways involved in photoreceptor differentiation and maintenance. In this direction, the miR-183 cluster targets PAX6 (Peskova et al., 2020), a highly conserved paired-box transcription factor that is critical for eye morphogenesis in a wide range of species (Glaser et al., 1992, 1994; Lauderdale et al., 2000; Davis et al., 2021).

The exact mechanisms that regulate the oscillatory expression of this miRNA cluster are not well understood. The putative promoter region of the miR-183 cluster contains several binding sites for transcription factors known to regulate the circadian rhythm in the eye, including RORα (Xu et al., 2007), but there is currently no experimental data to confirm this transcriptional regulation. Genetic variants with abnormal processing of pre-miR-182 have been described (Saus et al., 2010) and neuronal miRNAs have been shown to have very quick turn-over ratios (Krol et al., 2010) compared to nonneuronal cells (Bhattacharyya et al., 2006; Hwang et al., 2007; Krol et al., 2010). Thus, the regulation of miRNA processing and/or degradation could also play important roles in its oscillatory behavior.

A recent phenotype-driven genome-wide miRNA screen using reporter human cell lines identified several miRNAs with the potential to modulate circadian rhythms (Zhou et al., 2021). Among 989 miRNAs tested, this study identified 120 miRNAs that significantly changed the period length in a dose-dependent manner, including let-7, miR-17, and the miR-183 cluster. Importantly, these changes were tissue-specific and the inactivation of the miR-183 cluster shortened the circadian period in the retina but did not change the period length of the SCN in mice. All three members of the miR-183 cluster can modulate circadian rhythms and luciferase-based assays have shown that miR-182 potentially targets CLOCK (Saus et al., 2010) as well as the circadian rhythm regulators ADCY6 and MITF (Xu et al., 2007), while miR-96 directly targets PER-2 (Zhou et al., 2021) (Figure 3). Similarly, experimental evidence in zebrafish indicates that miR-183 targets other circadian regulators such as E4BP4-6 and AANAT2 (Ben-Moshe et al., 2014). However, these results do not exclude possible additional regulation through non-cell autonomous mechanisms.

Finally, while it is not known whether the oscillatory behavior of miR-183 has any effects on retinal development, the removal of circadian clock genes led to defective dorso-ventral patterning of cones, thinner inner retinal nuclear and plexiform layers, and reduced photoreceptor viability (Ait-Hmyed et al., 2013; Baba et al., 2018). Future studies might shed light on the role of the miR-183 cluster in these phenotypes and the interplay between the circadian rhythmicity and miRNA roles in photoreceptor differentiation and function.

### Let-7 Levels Oscillate With the Cell Cycle in the Embryonic Retina

**FIGURE 4:**
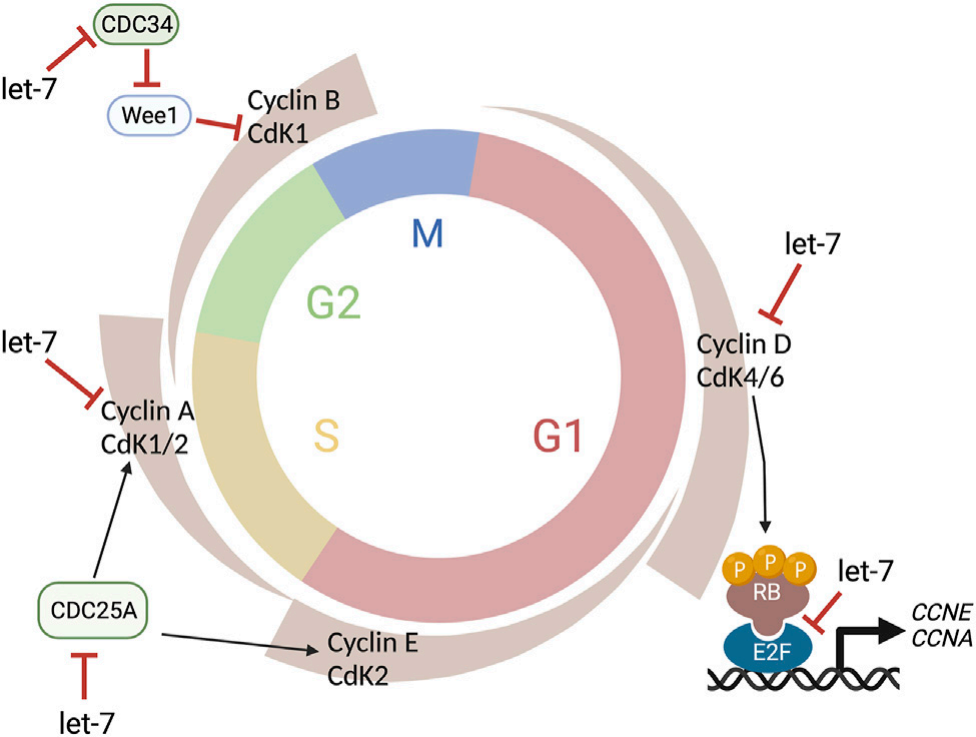
Let-7 regulation of the cell cycle. Let-7 regulates the cell cycle kinetics by both promoting cell cycle exit and lengthening distinct phases. Let-7 targets Cyclin D, CDK4, and CDK6, CDC25A, Cyclin A, and CDC34, affecting the G1/S, S/G2, and G2/M transitions, respectively. This figure was created with BioRender.com.

The cell cycle is a precisely regulated oscillatory process essential for growth and maintenance of tissues as well as for coordinating the timing of major cellular events during development. The cell cycle is classically divided into four different phases: Gap1 (G1), DNA Synthesis (S), Gap2 (G2), and Mitosis (M) (Norbury and Nurse, 1992). The ability of the cells to progress though these phases to ultimately produce two daughter cells is generally attributed to two classes of molecules: Cyclin-dependent kinases (CDKs), a large family of serine/threonine kinases, and their binding partners named Cyclins because their concentration varies in a cyclical manner (Malumbres and Barbacid, 2001). The abundance of individual Cyclins, and the consequent activation of the appropriate CDKs at specific phases, orchestrates the orderly completion of DNA replication and cell division and constitutes the core cell cycle oscillator (Figure 4). Thus, CyclinD/CDK4,6 activity ensures G1 progression, CyclinE/CDK2 promotes the G1/S transition, while CyclinA/CDK2 regulates the transition between S and G2. Finally, CyclinB/CDK1 warrants the G2/M transition and entry of cell into mitosis (Malumbres and Barbacid, 2001). However, cell cycle progression is not only regulated by the rise and fall of Cyclin molecules’ concentrations, but is tightly regulated at several levels and through many different mechanisms (Figure 4).

The first studies on miRNAs published three decades ago already suggested a role for the miRNA let-7 in the cell cycle of *C. elegans* (Ambros, 2001; Lee and Ambros, 2001). Let-7 is part of the heterochronic pathway required in the nematode seam cells to determine the timing of stage-specific developmental events (Ambros and Horvitz, 1984; Moss et al., 1997). Since then, numerous studies have revealed that let-7 is a master regulator of cell proliferation. Accordingly, let-7 alters cell cycle progression, controls the timing of cell cycle exit, and inhibits self-renewal, and disruptions in let-7 coding genes can enhance oncogenic transformation (Johnson et al., 2005; Sampson et al., 2007; Zhao et al., 2010).

Elegant genetic studies from the Ruvkun laboratory (Pasquinelli et al., 2000; Reinhart et al., 2000) revealed that let-7 is a genetic switch that controls major developmental transitions in bilaterally symmetrical animals, from flies and worms to vertebrates. In the developing mammalian retina, let-7 regulates the developmental transition that allows the retinal progenitors to generate the late cell types (amacrine cells, rod photoreceptors, bipolar cells, and Müller glia) (La Torre et al., 2013; Xia and Ahmad, 2016), and also plays a central role in Müller glia-dependent regeneration (Ramachandran et al., 2010; Wohl et al., 2019). Similar roles have been described in other parts of the developing CNS, where let-7 is required for the generation of the later cell populations in different species (Wu et al., 2012; Patterson et al., 2014; Shu et al., 2019).

In the developing retina, let-7 regulates cell cycle kinetics by both promoting cell cycle exit and lengthening S/G2 phases (Fairchild et al., 2019). Notably, no differences were detected in G1 length in time-lapse experiments using the fluorescent reporter FUCCI (Fluorescence Ubiquitination-based Cell Cycle Indicator) in combination with gain-of-function or loss-of-function of let-7 (Fairchild et al., 2019). Given that let-7 levels normally increase throughout developmental time in the retina (Arora et al., 2007; La Torre et al., 2013; Xia and Ahmad, 2016) (Table 1), these data correlate with classic experiments using ^3^H-thymidine cumulative labeling that indicated that the cell cycle lengthens during retinal development mainly due to an increase in S-phase length (Alexiades and Cepko, 1996).

The cell cycle proteins CDC25A, CDC34, CDK4, CDK6, Cyclin A, Cyclin D1, D2, and D3 are known let-7 targets (Bueno and Malumbres, 2011) (Figure 4) as well as TLX (Zhao et al., 2010), another cell cycle regulator, and oncogenic chromatin proteins such as HMGA1 and HMGA2 (Lee and Dutta, 2007; Xia and Ahmad, 2016). However, since the specific effects of let-7 overexpression or inhibition are different in different experimental paradigms (*e.g.*, induction of cell cycle arrest *vs* cell cycle lengthening), let-7’s ability to target these genes may be concentration and/or context dependent. Similarly, the human genome contains 10 different mature miRNAs in the let-7 family (let-7a, let-7b, let-7c, let-7d, let-7e, let-7f, let-7g, let-7i, mir-98, miR-202), produced from 13 precursor sequences. As each of these miRNAs have identical seed sequences and highly conserved regions for target recognition and thus, the individual roles and targets for each let-7 are not well characterized.

Strikingly, not only does let-7 regulate developmental transitions and cell cycle, but its expression and activities also oscillate concurrently with the cell cycle across the developing CNS, including the retina (Fairchild et al., 2019) (Figure 2B). Neural progenitors undergo interkinetic nuclear migration between the apical and basal surfaces in concert with the cell cycle (Sauer, 1935; Miyata, 2008; Norden et al., 2009). Thus, mitotic cell bodies are only found in the apical surface, and cell somas move basally in G1. Cells in S-phase are found at the most basal positions, which move again apically in G2. Intriguingly, let-7 levels also fluctuate within these regions, suggesting that let-7 oscillates in coordination with cell cycle (Fairchild et al., 2019). Mathematical modeling also supports that oscillatory levels of let-7 are required for the complex balance between let-7 and Cyclin/CDK complexes (Gerard et al., 2019) and more recently, these fluctuations have been validated by flow cytometry analyses and time-lapse imaging (Fairchild et al., 2019).

The cell cycle-dependent fluctuation of let-7 suggests that some cell cycle genes may be regulating its expression; however, given that the let-7 family is located in 13 different loci in the genome, the transcriptional regulation of these miRNAs is still poorly understood. E2F transcription factors have been shown to directly regulate let7a-d and let-7i expression and c-MYC represses the expression of several let-7 clusters (Bueno and Malumbres, 2011). Additionally, CyclinD1 can regulate the expression of Dicer (Yu et al., 2013) and thus, cell cycle-dependent miRNA processing may have an impact on let-7 fluctuations. Consistent with this idea of negative feedback loops, the miRNA machinery can be directly regulated by miRNAs, for example, a loop involving let-7 and Ago2 is critical to maintain pluripotency (Liu et al., 2021). Importantly, miRNA stability and turn-over rates could also be regulated in a cell cycle-dependent manner.

The specific role(s) of the periodicity of let-7 expression and activity have not been previously explored but it can be speculated that cell cycle-coupled miRNA oscillatory circuits may be an important strategy to coordinate division rates with complex cellular activities as well as the timing of cell cycle exit and fate decisions.

### MiR-9 Is Part of the Notch Ultradian Oscillator

Proper retina development relies on the tight balance between retinal progenitor cell (RPC) proliferation and differentiation. It is well-documented that Notch activation perpetuates RPC maintenance, whereas Notch pathway disruption leads to neuronal differentiation (Dorsky et al., 1995; Tomita et al., 1996; Jadhav et al., 2006; Nelson et al., 2007; Kaufman et al., 2019). Notch also regulates neural patterning (Baek et al., 2006; Bosze et al., 2020), cell fate specification (Yaron et al., 2006; Riesenberg et al., 2009; Chen and Emerson, 2021), is essential for Müller glia development (Furukawa et al., 2000; Bernardos et al., 2005; Nelson et al., 2011), and a key mediator of regeneration (Conner et al., 2014; Sahu et al., 2021). Together, a growing body of literature supports the notion that the Notch pathway is dynamic and remarkably pleiotropic, and that the timing and levels of Notch signaling must be precisely regulated to maintain the temporal control driving normal retinal development.

**FIGURE 5:**
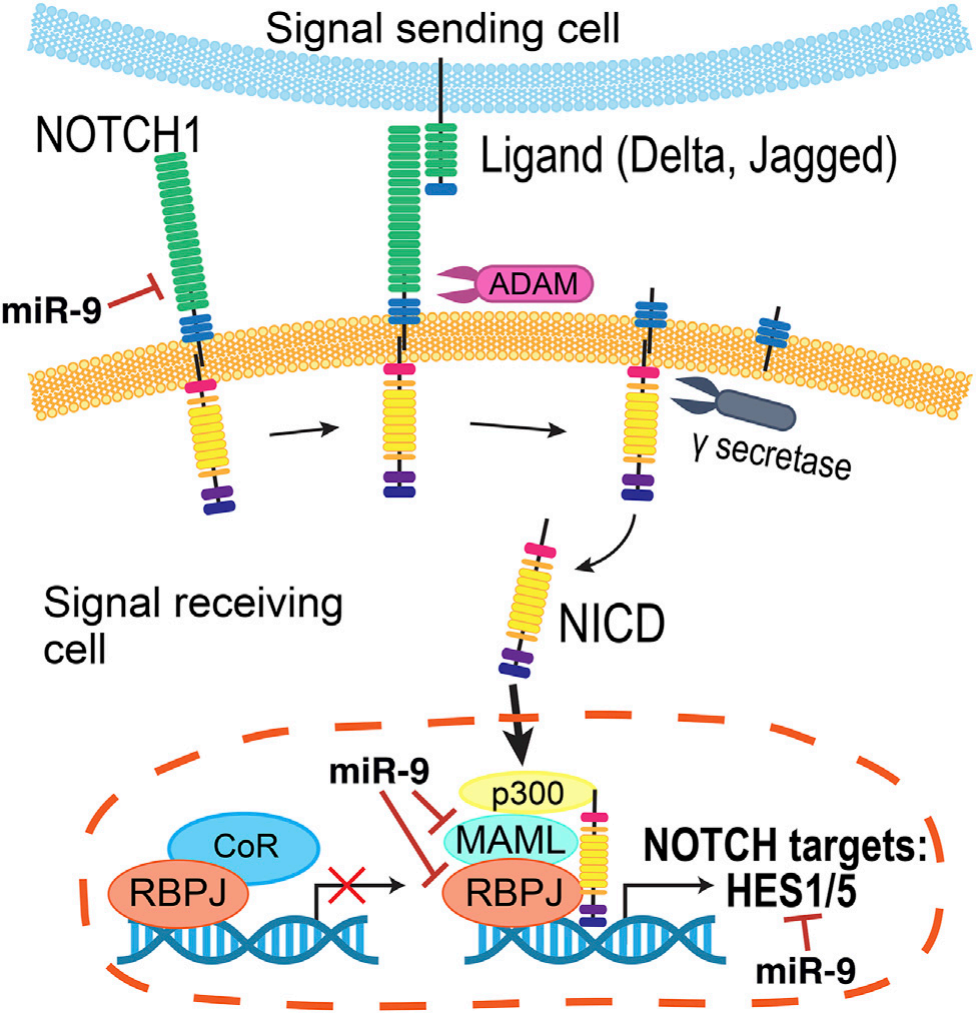
Summary of the Notch pathway. Notch signaling is initiated when a transmembrane Notch receptor (Notch1-4) on one cell is activated by a neighboring cell’s ligand (Delta, Jagged/Serrate), prompting proteolytic cleavage events by ADAM and γ-secretase to release the Notch receptor’s intracellular domain (NICD). Inside the cell, NICD translocates into the nucleus to form a transcriptional complex with a number of co-activators to in turn activate the expression of genes, including the Hes and Hey families. The miRNA miR-9 regulates several members of this pathway.

Since the Notch receptor was first identified in *Drosophila* over 100 years ago (Dexter, 1914), genetic and molecular interaction studies have helped map the Notch signaling pathway that is recognized today (Figure 5) (reviewed in Louvi and Artavanis-Tsakonas, 2006; Pierfelice et al., 2011; Bray, 2016). Briefly, the intracellular signaling pathway is initiated by cell-cell contacts, where the transmembrane Notch receptor (Notch1–4) on one cell is activated by a ligand (Delta-like (Dll1, Dll3, and Dll4), or Jagged (Jag1 and Jag2)) on a neighboring cell. Ligand binding prompts a series of proteolytic cleavage events that culminates in the release of the Notch receptor’s intracellular domain (NICD). NICD translocates into the nucleus, where it forms a transcriptional complex with Rbpj (recombination signal-binding protein for immunoglobulin kappa J region) and Maml1 (Mastermind-like transcriptional co-activator 1) to activate gene expression. The best characterized Notch targets are the Hes (Hes1, Hes3, and Hes5) and related Hey genes (Ohtsuka et al., 1999), which encode inhibitory basic helix-loop-helix (bHLH) proteins that suppress pro-neural bHLH genes Ngn1, Ngn2 (Neurogenins 1–2), NeuroD1, NeuroD2, NeuroD4, NeuroD6 (Neuronal Differentiation 1-2,4,6), and Ascl1 (Acheate-Scute) (Taylor et al., 2015; Dennis et al., 2019). Importantly, Hes proteins also repress the expression of Notch ligands, affecting the Notch activity of their neighbors (Jarriault et al., 1995).

These cross-regulatory activities raise a hypothetical problem: in the absence of Hes1, cells prematurely differentiate into neurons, but then how is the progenitor pool maintained if Hes1 reduces Notch signaling in neighboring cells? Several pioneering studies from the Kageyama laboratory solved the conundrum and showed that the expression of Hes1, Ascl1, and Dll1 display oscillatory behaviors (Figure 2C top) using luciferase-based reporters in several contexts (Masamizu et al., 2006), including neural progenitors (Shimojo et al., 2008; Imayoshi et al., 2013). These oscillatory expression patterns are driven by the Hes1 oscillator (Hirata et al., 2002). Hes1 protein represses its own expression by binding to N-box regulatory elements in the Hes1 promoter, and both Hes1 protein and mRNA have very short half-lives. Thus, upon repression, the levels of Hes1 decline rapidly, leading to the reactivation of Hes1 transcription with a rhythmicity of 2–3 h (Hirata et al., 2002). These oscillations are key in maintaining pools of progenitor cells from precociously differentiating; when Hes1 oscillations are quenched, even if the Notch signaling pathway can still be activated, neural progenitors undergo premature cell cycle exit (Shimojo et al., 2016). Importantly, these rhythmic patterns can in part explain the heterogeneity of gene expression observed in individual RPCs with “snapshot” techniques such as immunostaining and sequencing (Cepko, 2014; Dixit et al., 2014; Clark et al., 2019; Sridhar et al., 2020).

Many signaling pathways are common beneficiaries of miRNA-mediated regulation, and the Notch pathway is no exception (Inui et al., 2010; Roese-Koerner et al., 2016). In fact, functional relationships between Notch and miRNA pathways have been described in the developing retina as Dicer conditional knockout mice showed downregulation of Notch pathway components and at the same time, overexpression of NICD in Dicer-null retinas did not lead to classic “Notchy” phenotypes such as induction of glial fates (Georgi and Reh, 2011).

MiR-9, a miRNA highly expressed in the developing CNS, interacts with Notch components in several organisms (Tan et al., 2012). Target prediction analyses have shown that miR-9 may directly target components of the Notch pathway, including Notch ligands, Rbpj, and Maml1 (Roese-Koerner et al., 2017). Additionally, bioinformatics analyses have identified miR-9-binding sites in mouse, rat, and human *Hes1* (Baek et al., 2006), zebrafish *her5* and *her9* (Leucht et al., 2008), and Xenopus *hairy1* (Bonev et al., 2011). Manipulation of miR-9 activity by antisense inhibitors resulted in increased levels of Hes1, and overexpression of miR-9 conversely reduced the half-life of Hes1 (Bonev et al., 2012).

**FIGURE 6:**
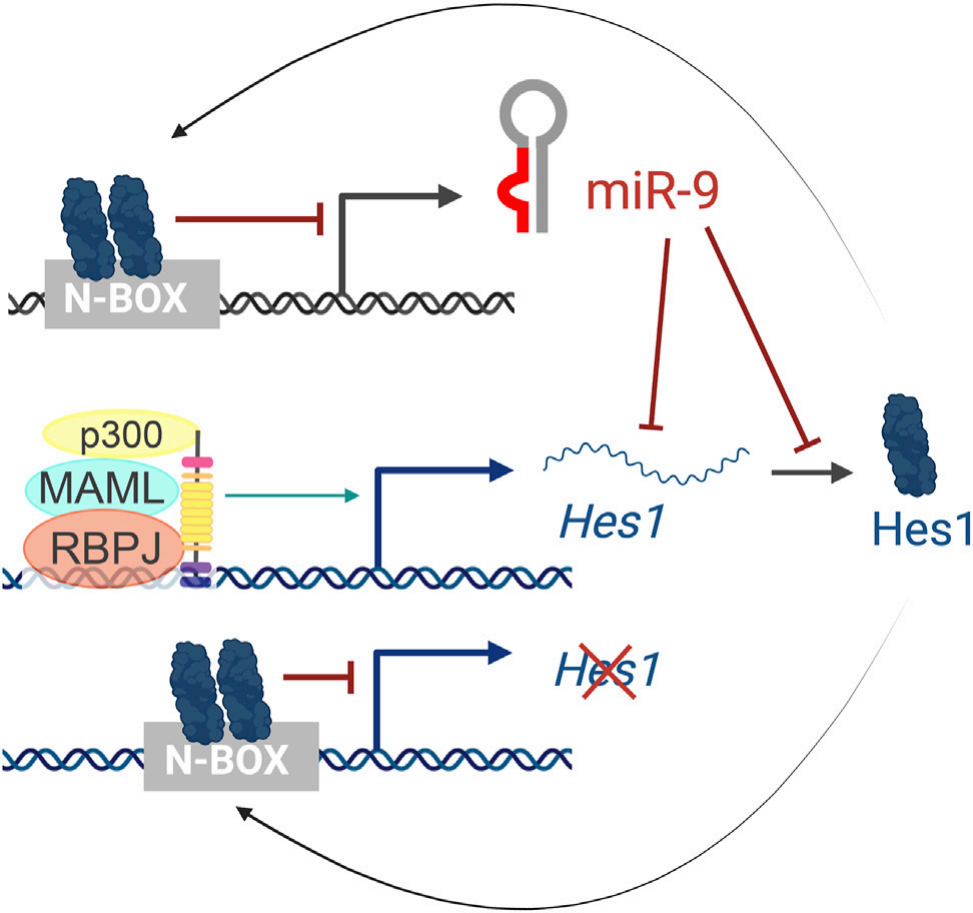
The Hes1/miR-9 oscillator. Activation of the Notch pathway leads to the activation of Hes1 transcription. Hes1 protein then dimerizes and binds to N-box domains to repress its own expression as well as miR-9 transcription. In turn, miR-9 reduces Hes1 levels by controlling the stability of *Hes1* mRNA and inhibiting its translation, resulting in oscillatory behaviors.

In humans, miR-9 is transcribed from three independent genomic loci (pri-miR-9-1, pri-miR-9-2, and pri-miR-9-3) that give rise to two functional miRNAs, miR-9-5p and miR-9-3p. Hes1 reduces miR-9 expression, as observed by *in situ* hybridization of pri-miR-9-2 in the mouse cortex and binds to several N-boxes in the putative miR-9 promoters of all three miR-9 genes (Figure 6). However, Hes1 only regulates the expression of pri-miR-9-1 and pri-miR-9-2, but not pri-miR-9-3 (Bonev et al., 2012). These promoters are embedded within CpG islands and thus, they could also be regulated by epigenetic mechanisms or other indirect means.

Importantly, the cross-regulations between miR-9 and Hes1 (Figure 6) also contribute to the Hes1 oscillator, and overexpressing or inhibiting miR-9 has been shown to reduce Hes1 oscillations (Bonev et al., 2012). The negative feedback loops between miR-9 and Hes1 creates an out-of-phase oscillatory pattern of expression (Figure 2C top), which is important for limiting Hes1 oscillations (Roese-Koerner et al., 2016). As development continues, miR-9 accumulates (due to its longer half-life than the less-stable Hes1 mRNA and protein) until it reaches a threshold for differentiation (Shimojo et al., 2016). At that point, Hes1 oscillations are dampened, and miR-9 maintains high, steady levels allowing for neural differentiation to proceed (Figure 2C bottom) (Bonev et al., 2012). Although oscillations of miR-9 have not been observed directly, mathematical modelling analyses incorporating miR-9 into the Hes1 oscillator recapitulate the behaviors observed experimentally (Goodfellow et al., 2014).

During retinal development, miR-9 expression increases in RPCs over developmental time and regulates cell fate acquisition (La Torre et al., 2013). Mir-9 is also important in the mature mouse retina to maintain homeostasis of the Müller glia (Wohl et al., 2017) and can potentiate Müller glia conversion into progenitor-like cells in culture in combination with miR-124 (Wohl and Reh, 2016b). While the molecular mechanisms downstream of these functional roles remain widely unexplored, it can be speculated that the oscillatory interplay between Notch and miR-9 in combination with the increasing levels of mature miR-9 over time may be one of the mechanisms that enables cells with an ability to keep track of time while maintaining the ability to adapt to external stimuli. This model accommodates the existing evidence indicating that fate decisions during retinal development are both cell autonomous and strongly influenced by external factors.

Recently, a novel CIS-regulatory element of pri-miR-9-2 has been described for its association with retinal diseases such as Macular Telangiectasia Type 2 and Macular Degeneration (Thomas et al., 2021). Interestingly, the deletion of this enhancer leads to reduced miR-9 levels, a decrease in the number of rod photoreceptors, and perturbation of Müller glia homeostasis in human retinal organoids. Transcriptional data indicates that the Notch pathway is affected in these cells, but the exact nature of this regulation and whether the oscillatory behaviors and feedback loops between Notch and miR-9 play any roles have not yet been investigated.

## Conclusions and Perspectives

During normal development, different cell fates are specified with exquisite spatial and temporal accuracy. Oscillatory feedback circuits that integrate temporal cues are part of the machinery that establishes the robustness of developmental transitions and progenitor outcomes. It is now obvious that miRNAs are functionally integrated in many of these oscillatory pathways. Beyond the examples offered in this review, a reciprocal relationship between miRNAs and transcription factors that in turn regulate miRNA expression may be a common theme in a variety of developmental contexts.

Despite all the evidence accumulated in the last few years, we are only starting to understand the relevance of these rhythmic behaviors, largely because most of the miRNA expression data to date comes from studies that used sequencing technologies that do not capture dynamic changes of expression within a cell. Thus, efforts to develop tools to show miRNA levels longitudinally with cellular resolution need to be advanced.

In addition, our understanding of miRNA transcriptional regulation is still quite limited. The complex regulation of miRNA processing and turn-over may similarly open new avenues to further understand the regulatory networks that govern neural development.
